# *γ*-Aminobutyric acid treatment enhances quality and improves antioxidant activities of fresh-cut *Euryale ferox* stems during postharvest storage

**DOI:** 10.3389/fnut.2025.1527555

**Published:** 2025-02-27

**Authors:** Yan Wang, Hongmei Wei, Songxu Wang, Fulong Yu, Ligong Zhai, Jianting Yang, Zhaohui Wei

**Affiliations:** College of Food Science and Engineering, Anhui Science and Technology University, Anhui, China

**Keywords:** fresh-cut *Euryale ferox* stem, *γ*-aminobutyric acid, antioxidative activities, postharvest storage, quality

## Abstract

**Introduction:**

Fresh-cut *Euryale ferox* stem (FCEFS) is a nutrient-rich but underutilized vegetable that has a short shelf life, making it prone to softening and rotting.

**Methods:**

The effects of different concentrations of *γ*-aminobutyric acid (GABA) on sensory quality and antioxidant activity during cold storage at 4°C for 20 d were examined.

**Results:**

The results revealed that the FCEFS samples treated with 5 mM GABA maintained greater hardness and ascorbic acid, total phenolic compound and chlorophyll contents than the FCEFS samples not treated with GABA. FCEFS samples subjected to GABA treatment and stored under long-term cold storage conditions presented elevated sensory scores. The control group had ceased to possess commercial value on 16 d, and it was edible by 20 d following GABA treatment, which was corroborated by the notable colour characteristics and electronic nose analysis. Notably, GABA treatment delayed the decrease in soluble solid and endogenous GABA levels, and delayed the accumulation of malondialdehyde and the increase in lignin content in FCEFS during cold storage. In addition, GABA maintained high peroxidase (POD), superoxide dismutase (SOD), ascorbate peroxidase (APX) and phenylalanine ammonia lyase (PAL) activities.

**Discussion:**

Treatment with 5 mM GABA maintained the shelf-life and sensory quality of the FCEFS for 20 d at 4°C. Therefore, these results indicate that GABA can be used to delay the softening of FCEFS and extend its cold storage and shelf-life.

## 1 Introduction

Fresh fruits and vegetables continue to undergo physiological and metabolic processes even after harvesting, which can lead to quality issues such as browning, softening, rotting, and nutrient loss during transport, storage, and marketing. These issues result in significant economic losses (1). Researchers worldwide have explored various chemical and physical methods to delay senescence in fruits and vegetables. Refrigeration is a primary method used to extend shelf-life and maintain quality by delaying the ageing process postharvest (2). The goal of these methods is to maintain the scavenging capacity of reactive oxygen-metabolizing enzymes, thereby preserving product quality.

*Euryale ferox*, a member of the water lily genus *Euryale*, is an annual aquatic herbaceous plant that thrives in temperate and tropical regions. Fresh-cut *Euryale ferox* stem (FCEFS) is rich in dietary fibre, polyphenols, and polysaccharides (3). The FCEFS includes foods that satisfy modern consumer preferences for natural and healthy options. However, the brittle texture and high moisture content of FCEFS pose significant challenges for storage. Consequently, most of these FCEFS are relegated for use as animal feed, which significantly reduces their edibility and results in economic losses during storage, transport, and sale. Research has shown that 1-MCP treatment can help maintain better appearance quality and higher antioxidant content in fox nuts, thereby enhancing their refrigerated storage quality. The available preservation methods for FCEFS are limited. Therefore, methods to retard ageing effectively, reduce the respiration rate, and improve the sensory quality of FCEFS have become popular research topics.

GABA is a nonprotein amino acid found in animals, plants, and microorganisms (4). As a novel functionally active factor, GABA has been explored as a food additive. Numerous studies have been conducted on the exogenous application of GABA, as a postharvest treatment for fruits and vegetables, to reduce the effects of biotic and abiotic stresses. Research indicates that exogenous GABA enhances the accumulation of endogenous GABA in apples, increases enzyme activity and gene expression related to oxygen metabolism, and improves disease resistance in apple fruits (5). Shekari et al. (6) reported that 0.1 mM GABA treatment led to a reduction in the accumulation of reactive oxygen species and a decrease in membrane peroxidation in *Agaricus bisporus*. Furthermore, GABA has been shown to decrease the hydrogen peroxide content and increase ascorbic acid content. Additionally, the application of GABA significantly increased the contents of total phenols, total flavonoids, flavanols, and carotenoids (7). In addition, GABA exhibits ROS scavenging activity and possesses the ability to stimulate the antioxidant activities of the treated fruits (2). It was reported that GABA could protect cell membranes, alleviate the damage caused by oxidative stress to delay senescence in kiwifruits (8).

Although reports have indicated that GABA can be used to extend the storage life of fruits and vegetables, the effects of GABA treatment on FCEFS during cold storage have not been studied. Therefore, this study examines the impact of exogenous GABA application on quality preservation, oxidative stress, lipid peroxidation, and antioxidant activity in FCEFS after cold.

## 2 Materials and methods

### 2.1 Materials

#### 2.1.1 Samples

**Figure 1:**
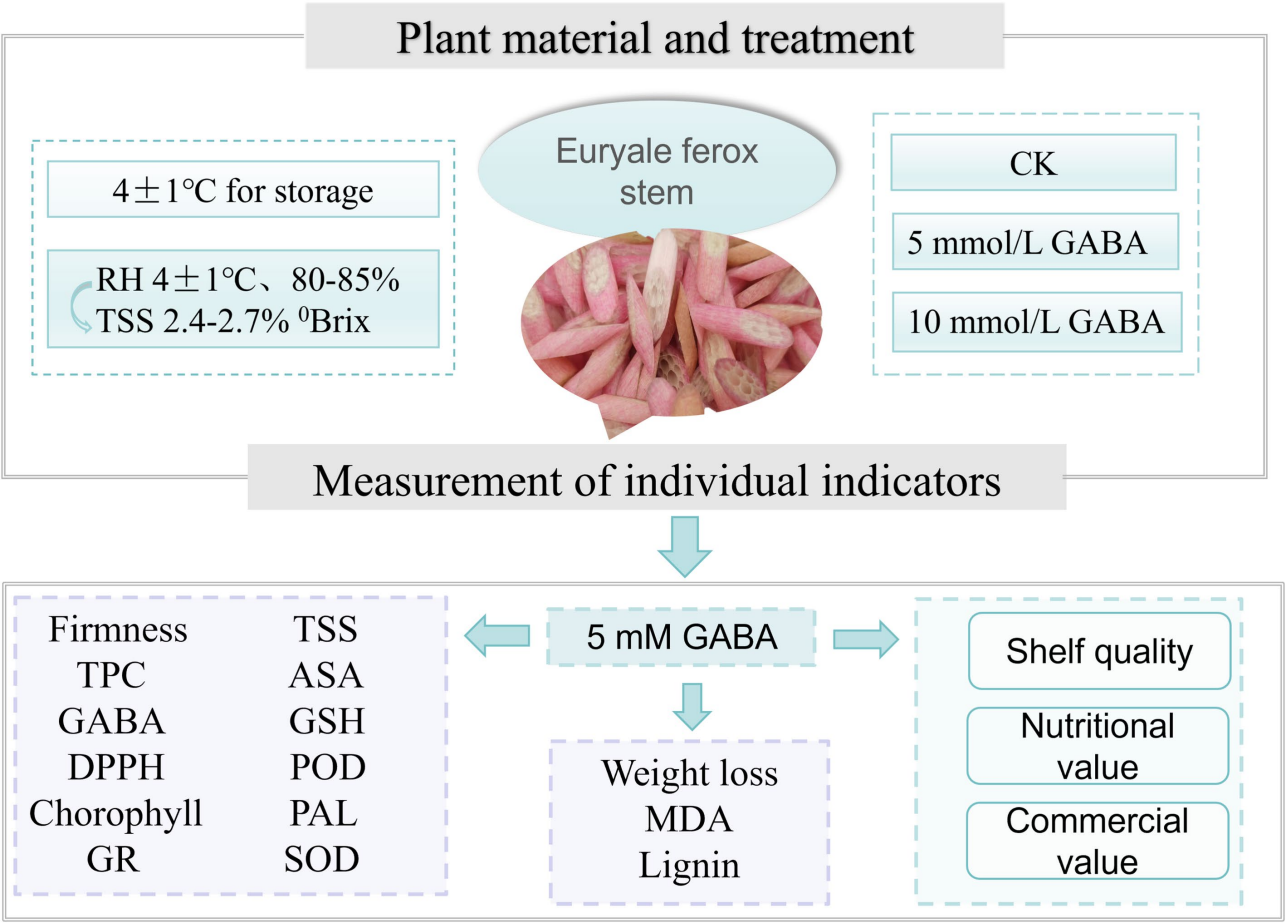
Experimental design and procedure of this study. Control: deionized water treatment; different concentrations of GABA were used for treatment.

*Euryale ferox* stems were sourced from the planting base of Xiangpiaopiao Agricultural Products Development Co., Ltd., located in Fengyang County, Chuzhou city, Anhui Province, China. The *Euryale ferox* stems were similar in terms of maturity, with total soluble solid (TSS) contents ranging from 2.4–2.7%. Within 2 h of harvest, the stem samples were transferred to the laboratory and precooled for 30 min. Uniformly sized, evenly thick, disease-free, and nondamaged *Euryale ferox* stems were selected, surface sterilized with 0.1% sodium hypochlorite for 3 min, washed, peeled, and sliced into 4 mm thick pieces. The preliminary experiments employed GABA concentrations of 5 mM and 10 mM. Subsequent trials used a 5 mM concentration of GABA, as it proved to be the most effective in preserving the quality of FCEFS. The FCEFS were then randomly assigned to two groups, each weighing approximately 3 kg. Samples of the sliced FCEFS was submerged in 5 mM GABA solution for 15 min, whereas the control samples underwent the same process in distilled water. After soaking, the samples from both groups were drained in a biosafety cabinet and then immediately packed into PE bags (18*20 cm), with each bag containing 150 g of sample. The samples were stored at 4°C with 85–90% relative humidity, and assessments were made on days 0, 4, 8, 12, 16, and 20 d. Simultaneously, a homogenized composite sample was prepared using liquid nitrogen and stored at −80°C until further analysis (Figure 1).

#### 2.1.2 Instrument equipment

The NR110 colorimeter was purchased from 3nh Technology Co., Ltd., in China; AIRSENSE Electronic Nose PEN 3 was produced by Beijing Ensoul Technology Co., Ltd., in China; the UV-1800 ultraviolet–visible spectrophotometer was purchased from Shimadzu, Kyoto, Japan; and the EVO-18 scanning electron microscope was produced by Carl Zeiss AG, Germany.

### 2.2 Firmness, weight loss rate, and colour

The firmness of the FCEFS was measured via a texture analyser. A probe with a diameter of 4 mm was pressed to a depth of 2 mm at the equatorial region of each stem. The maximum force developed during the test was recorded and expressed in Newtons (N). Ten samples were collected, and the measurements were taken at the symmetrical part of each sample.

The weight loss rate of the FCEFS was calculated via the weighing method. The weight loss was calculated from the initial weight and the weight on each sampling day, and the formula was as follows (Equation 1):


(1) $$\begin{array}{l} \mathrm{Weight loss}\;\left(\%\right)=[(\mathrm{initial weight}-\mathrm{weight in each}\\ \;\mathrm{sampling}\;\mathrm{day})/\mathrm{initial weight}]\times 100\% \end{array}$$

The colour of the FCEFS was measured via a calibrated colorimeter at symmetrical points on both sides of the equatorial part of each fruit. The total colour difference (ΔE) was calculated from the colour at each sampling point during storage (L*, a*, b*) and the initial colour at the start of storage (L_0_*, a_0_*, b_0_*), as described by Duangmal et al. (9), according to the following formula (Equation 2):


(2) $$\Delta E={\left[{\left(L^{*}-L_{0}^{*}\right)}^{2}+{\left(a^{*}-a_{0}^{*}\right)}^{2}+{\left(b^{*}-b_{0}^{*}\right)}^{2}\right]}^{1/2}$$

### 2.3 Appearance, sensory evaluation and electronic nose analyses

The FCEFS samples were treated with varying concentrations of GABA in PE bags. Every four days, their appearance was observed and photographed, and any changes in visual characteristics were documented. Sensory evaluation was conducted via the method described by Jia et al. (10) and involved 5 trained men and 5 trained women. The evaluation criteria included the visual appearance, colour, freshness, odour, acceptability and general evaluation of the FCEFS (Table 1). For each treatment, 8 samples were placed on a white board for observation and evaluation.

**Table 1:** Sensory evaluation rating scale.

score	Visual appearance	Color	Odour	Freshness	Acceptability	General evaluation
10–7	Exemplary	Red and glossy	Exemplary	Exemplary	Excellent, no defects	Excellent
7–5	Good	Red	Good	Small loss	Very good	Good
5–2	Fair	Local yellow	Loss of fresh fragrance	Further loss	Limited edible quality	Fair
2–0	Poor	Severe yellow	Obvious foul odour	Serious water loss	Inedible	Poor

An electronic nose was used to analyse the odour of the FCEFS samples via the method described by Zhu et al. (11), with some modifications. When 10 different metal oxide sensors were used as the sensor array, the sensor response time was less than 1 s. Three grams of each sample were ground, placed in a 10 mL vial and allowed to stand at room temperature for 2 h. The detection conditions were as follows: interval time of 1 s, air cleaning time of 70 s, zeroing time of 5 s, preinjection time of 5 s, test time of 120 s, and sensor chamber flow rate and initial injection flow rate of 400 mL/min. The data were measured three times for analysis in each treatment group.

### 2.4 Total soluble solid, chlorophyll, and ascorbic acid levels

The total soluble solid (TSS) content was determined via a digital refractometer.

The chlorophyll content was determined following the method described by Chen et al. (12), with slight modifications. Briefly, 1 g of tissue was added to a small amount of calcium carbonate powder and 3 mL of 95% (v/v) aqueous ethanol. The mixture was ground into a pulp and transferred to a 10 mL centrifuge tube. The sample was then centrifuged at 6000 × g for 15 min at 4°C. The precipitate was discarded, and the supernatant was adjusted to a final volume of 10 mL with 95% ethanol. A 95% ethanol solution was used as a blank control.

The ascorbic acid (AsA) content in the FCEFS samples was determined via the use of 2,6-dichlorophenol indophenol, following the method reported by Ali et al. (13). The AsA content is expressed as grams per kilogram (g/kg) of fresh weight (FW).

### 2.5 Total phenolic content and DPPH scavenging capacity

The Folin–Ciocalteu method was used to determine the total phenol content following the protocol described by García‐Pastor et al. (14). Briefly, 1 g of powder was homogenized in 5 mL of ethanol solution, and subjected to ultrasonic treatment for 30 min at 30°C. The mixture was then centrifuged to obtain the supernatant, which was stored at −20°C. For the reaction, 100 μL of Folin–Ciocalteu reagent, 100 μL of supernatant, and 0.2 mL of Na_2_CO_3_ were mixed. The reaction was carried out in the dark at 25°C for 60 min. The absorbance was measured at 760 nm, and the total phenol content was calculated using the standard curve prepared for gallic acid.

The DPPH scavenging capacity was evaluated as previously described (15), with slight modifications. A 0.2 g sample was mixed with 5 mL of 50% ethanol and ground to obtain a homogenate. This mixture was centrifuged at 12000 × g for 20 min at 4°C, and the supernatant was collected. The reaction system consisted of 0.1 mL of supernatant and 2.9 mL of 200 μM DPPH. After incubation in the dark at 25°C for 30 min, the absorbance was measured at 517 nm. The sample absorbance value was recorded as V_1_, and the control absorbance value was recorded as V_0_, with a DPPH ethanol solution used as the control. The DPPH scavenging capacity was calculated via the following formula and expressed as a percentage (Equation 3):


(3) $$\mathrm{DPPH scavenging capacity}\left(\%\right)=\left[\left(V_{1}-V_{0}\right)/V_{1}\right]\times 100\%$$

### 2.6 Malondialdehyde content, H_2_O_2_ content, lignin content and lignin staining

The malondialdehyde content was determined as described previously (16) with slight modifications. The crude extract samples were mixed with 0.6 mL of 0.5% thiobarbituric acid and heated at 95°C for 30 min. The mixture was cooled and then centrifuged at 10,000 × g for 10 min at 25°C. The absorbance was then measured at 532 nm and 600 nm with a spectrophotometer. The results are expressed in nmol/L FW.

A 0.3 g tissue sample was combined with 2.7 mL of PBS for extraction, followed by homogenization in an ice bath and collection of the supernatant. The reaction solutions included 0.1 mL of the obtained supernatant, 20 mol/L titanium tetrachloride and 1 mL of concentrated ammonia. The resulting precipitate was then dissolved in 1.0 mL of sulfuric acid solution, and the absorbance of the resulting solution was measured at a wavelength of 405 nm. The content of hydrogen peroxide was expressed in terms of mmol/g FW.

The lignin content was determined via a lignin content detection kit (Solarbio Biotechnology Co., Ltd.). The samples were dried at 80°C and ground to a constant-weight powder. The powder was passed through a 30–50 mesh sieve. Acetylated lignin was prepared in accordance with the kit instructions; based on the characteristic absorption peak of lignin acetylation products at 280 nm, a positive correlation was observed between the light absorption value and lignin content. The lignin content is expressed in mg/g.

The equatorial surface of the FCEFS samples was cut into thin slices with a thickness of approximately 2–3 mm, and the samples were soaked in 75% alcohol for 6 h. The rinsed slices were soaked in an alcohol solution containing 2% resorcinol (w/v) for 3 min, after which the slices were removed and drained, placed into a glass Petri dish, and treated with an appropriate amount of concentrated hydrochloric acid for 2 min. The lignin deposition site quickly turned pink and was observed under a light microscope.

### 2.7 Glutathione reductase and reduced glutathione

A 0.1 g sample was added to 1 mL of extract solution, followed by homogenization in an ice bath. The mixture was then centrifuged at 8,000 × g for 10 min at 4°C to obtain the supernatant.

Glutathione reductase activity was assayed according to the protocol described by Chumyam et al. (17). Briefly, the oxidation of NADPH by GSH was monitored spectrophotometrically at 340 nm. One unit of GR activity was defined as the amount of enzyme that catalyses the oxidation of one nanomole of NADPH per gram of sample per minute (nmol/min/g FW).

The GSH assay substrate consisted of 100 μL of the supernatant, 200 μL of 4 mmol/L 5,5′-dithiobis (2-nitrobenzoic acid), and 700 μL of 0.1 M phosphate buffer (pH 8.0). The absorbance of each sample was measured at 412 nm.

### 2.8 Endogenous GABA content

The endogenous GABA content was determined via the method described by Hu et al. (18). A 0.1 g sample was added to 1 mL of extraction solution and homogenized vigorously. The mixture was then heated in a water bath at 95°C for 2 h, followed by centrifugation at 8,000 × g for 10 min. The supernatant was mixed with 150 μL of potassium hydroxide, 120 μL of 6% phenol solution, and 180 μL of sodium hypochlorite and subsequently boiled for 10 min. The reaction was terminated by adding 600 μL of 60% ethanol. The absorbance was measured at 640 nm, and the GABA content is expressed in mg/g FW.

### 2.9 POD enzyme activity, SOD activity, APX activity and PAL activity

The activities of POD, SOD, APX and PAL were determined via various methods. Frozen powder tissue (0.1 g) was dissolved in different precooled buffers, homogenized, and centrifuged at 8000 × g for 20 min at 4°C. The supernatants were used for the respective enzyme activity assays.

POD enzyme activity was quantified following the method of Terefe et al. (19), with some modifications. The POD working solution comprised sodium phosphate buffer, H_2_O_2_, and 2% guaiacol solution. Fifty microlitres of enzyme extract was mixed with 950 μL of the working solution, and the absorbance of the reaction mixture was measured at 470 nm using a UV spectrophotometer.

SOD enzyme activity was determined according to previous work (20). The reaction system consisted of 800 μL of working solution, 50 μL of diluted SOD sample, 100 μL of xanthine nucleotide, and 50 μL of crude enzyme solution. The mixture was incubated at room temperature for 30 min, and the absorbance was measured at 450 nm.

The APX activity was determined according to the method of Shi et al. (21), with slight modifications. The reaction mixture consisted of phosphate buffer, hydrogen peroxide, and crude enzyme extract. The absorbance was measured at 290 nm.

PAL activity was determined by using the assay of Toscano et al. (22) with slight modifications. A 0.1 g sample of FCEFS was added to 0.9 mL of extraction solution, homogenized in an ice bath, and centrifuged at 10000 × g for 10 min at 4°C to obtain the supernatant. The reaction mixture included 40 μL of crude enzyme solution, 1,480 μL of buffer solution and 400 μL of phenylalanine solution. The mixture was incubated in a water bath at 30°C for 30 min, and the reaction was terminated by adding 80 μL of hydrochloric acid solution. The absorbance was measured at 290 nm.

### 2.10 Microstructure determination

Following the method described by Chi et al. (23), the FCEFS samples were cut into small square pieces with a volume of 1 mm^3^. The samples were dehydrated with ethanol and freeze-dried. After drying, the samples were placed on conductive carbon film double-sided adhesive and transferred to the sample stage of an ion sputtering instrument for gold coating. The microstructures of the samples were then observed via cold-field emission scanning electron microscopy with an accelerating voltage of 20.0 kV.

### 2.11 Statistical analysis

All the experiments and assays utilized three biological replicates, and the data provided in this study represent the mean ± standard deviation. The data were analysed using one-way analysis of variance (ANOVA) followed by Duncan’s multiple range test using the SPSS 27 statistical software program (SPSS Inc., Chicago). *p* < 0.05 was considered statistically significant.

## 3 Results

### 3.1 Firmness, weight loss and colour

**Figure 2:**
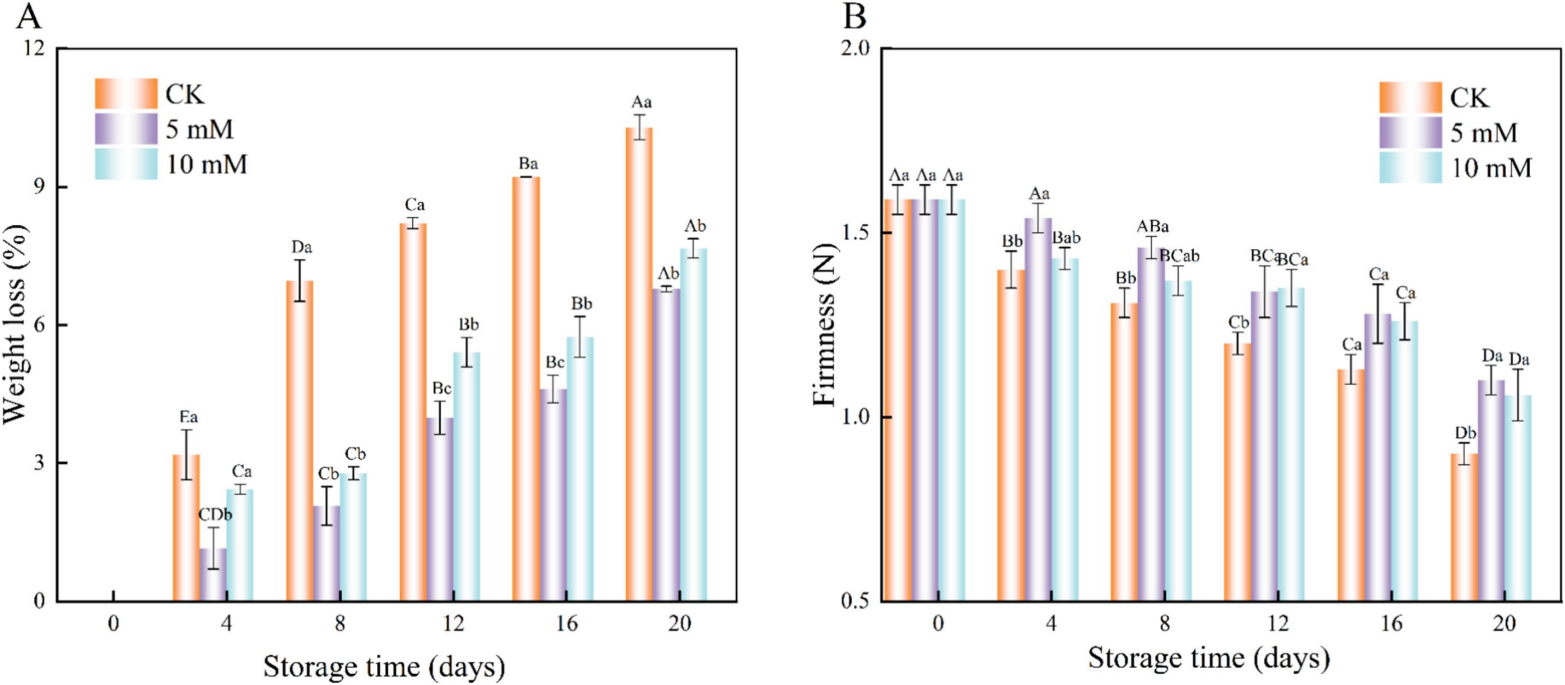
Interaction effect of the postharvest GABA content and storage time on the weight loss **(A)** and firmness **(B)** of the FCEFS samples stored at 4°C and 85–90 RH. The data are the means ± SDs (*n* = 3). Values with different lowercase letters at the same time point are significantly different (*p* < 0.05) between treatments. Values with different uppercase letters represent significant differences (*p* < 0.05) within a treatment at different sampling time points.

The interaction effect of storage time and treatment influenced weight loss. Throughout the storage period, the weight loss gradually increased over time (Figure 2A). However, the weight loss in the 5 mM GABA-treated samples was consistently lower than that in the control samples, with significant differences observed (*p* < 0.05). The firmness of the FCEFS also decreased over time across all the treatments, but the firmness of the GABA-treated samples was greater than that of the control samples (Figure 2B). After 20 d of storage, the firmness values of the control, 5 mM and 10 mM GABA-treated samples were 0.9 N, 1.1 N and 1.06 N, respectively, which were 0.69 N, 0.49 N, and 0.53 N lower than their initial values. This study revealed significant differences between the control group and the treatment group except on the 16th day (*p* < 0.05). GABA treatment can delay the decrease in firmness of the FCEFS.

Colour is a crucial quality attribute for evaluating the visual appeal of FCEFS, with smaller total colour difference values indicating lower degrees of browning. The L^*^ value of the FCEFS samples decreased during the storage period, indicating gradual darkening (Table 2). Compared with those of the control samples, the L^*^ values of the GABA-treated samples decreased more slowly. Among them, the L^*^ value of the 5 mM GABA treatment significantly differed at 20 d (*p* < 0.05). Overall, the a^*^ and b^*^ values also slowly decreased over the storage period. At 20 d, the a^*^ values were markedly lower in the 5 mM and 10 mM treatment groups than in the untreated control group.

**Table 2:** Interaction effect of the postharvest GABA content and storage time on the colour difference values L^*^, a^*^, b^*^ and ΔE of postharvest FCEFS stored at 4°C and 85–90 RH.

	Treatment			
	Storage time (days)	CK	GABA 5 mM	GABA 10 mM
L^*^	0	51.67 ± 1.15^Aa^	51.67 ± 1.15^Aa^	51.67 ± 1.15^Aa^
	4	51.23 ± 1.31^Aa^	51.48 ± 2.06^Aa^	49.77 ± 0.63^ABa^
	8	47.8 ± 1.12^Ba^	50.52 ± 1.28^ABa^	48.52 ± 0.49^BCa^
	12	47.56 ± 0.13^Ba^	49.52 ± 1.44^ABa^	47.7 ± 0.2^BCa^
	16	44.74 ± 1.04^BCb^	48.66 ± 0.15^ABa^	46.61 ± 1.51^Cab^
	20	43.37 ± 0.68^Cb^	46.78 ± 1.03^Ba^	45.95 ± 0.17^Cab^
a^*^	0	5.5 ± 0.29^Ba^	5.5 ± 0.29^Ba^	5.5 ± 0.29^Aa^
	4	6.14 ± 0.18^Aa^	6.14 ± 0.15^Aa^	5.38 ± 0.05^Ab^
	8	4.46 ± 0.14^CDa^	4.5 ± 0.07^Ca^	3.48 ± 0.17^Bb^
	12	3.98 ± 0.15^Da^	3.83 ± 0.13^Da^	3.01 ± 0.16^Bb^
	16	4.64 ± 0.13^Ca^	3.97 ± 0.03^Db^	3.07 ± 0.16^Bc^
	20	4.21 ± 0.07^CDa^	3.59 ± 0.02^Db^	3.49 ± 0.02^Bb^
b^*^	0	16.54 ± 0.22^Aa^	16.54 ± 0.22^Ba^	16.54 ± 0.22^Aa^
	4	16.26 ± 0.29^ABa^	17.41 ± 0.46^Aa^	16.95 ± 0.37^Aa^
	8	16.04 ± 0.03^ABa^	16 ± 0.12^Ba^	15.71 ± 0.22^Aa^
	12	15.82 ± 0.23^ABa^	15.6 ± 0.09^Ba^	15.57 ± 0.75^Aa^
	16	15.89 ± 0.29^ABa^	15.97 ± 0.34^Ba^	15.89 ± 0.47^Aa^
	20	15.72 ± 0.05^Ba^	15.93 ± 0.31^Ba^	15.83 ± 0.42^Aa^
∆E	0	0	0	0
	4	1.65 ± 0.23^Ea^	1.34 ± 0.33^Da^	2.1 ± 0.46^Da^
	8	4.49 ± 0.02^Da^	2.81 ± 0.29^Cc^	3.59 ± 0.02^Cb^
	12	5.55 ± 0.14^Ca^	3.29 ± 0.11^BCc^	4.52 ± 0.24^Bb^
	16	6.96 ± 0.24^Ba^	3.75 ± 0.12^Bc^	5.57 ± 0.29^Ab^
	20	8.56 ± 0.33^Aa^	5.98 ± 0.12^Ab^	6.25 ± 0.06^Ab^

Values with different lowercase letters at the same time point are significantly different (*p* < 0.05) between treatments. Values with different uppercase letters represent significant differences (*p* < 0.05) within a treatment at different sampling time points.

The total colour difference (ΔE) increased over time, with higher values indicating a greater deviation from the colour of fresh samples. Throughout the storage period, the ΔE values for the 5 mM GABA-treated samples were consistently lower than those of the control samples, indicating significant differences (*p* < 0.05). In summary, the L*, a*, and b* values tended to decrease during storage, indicating a decrease in brightness and a reduction in red and yellow colouration. The 5 mM GABA treatment was the most effective at maintaining the quality of FCEFS during storage.

### 3.2 Appearance, sensory evaluation and electronic nose analyses

**Figure 3:**
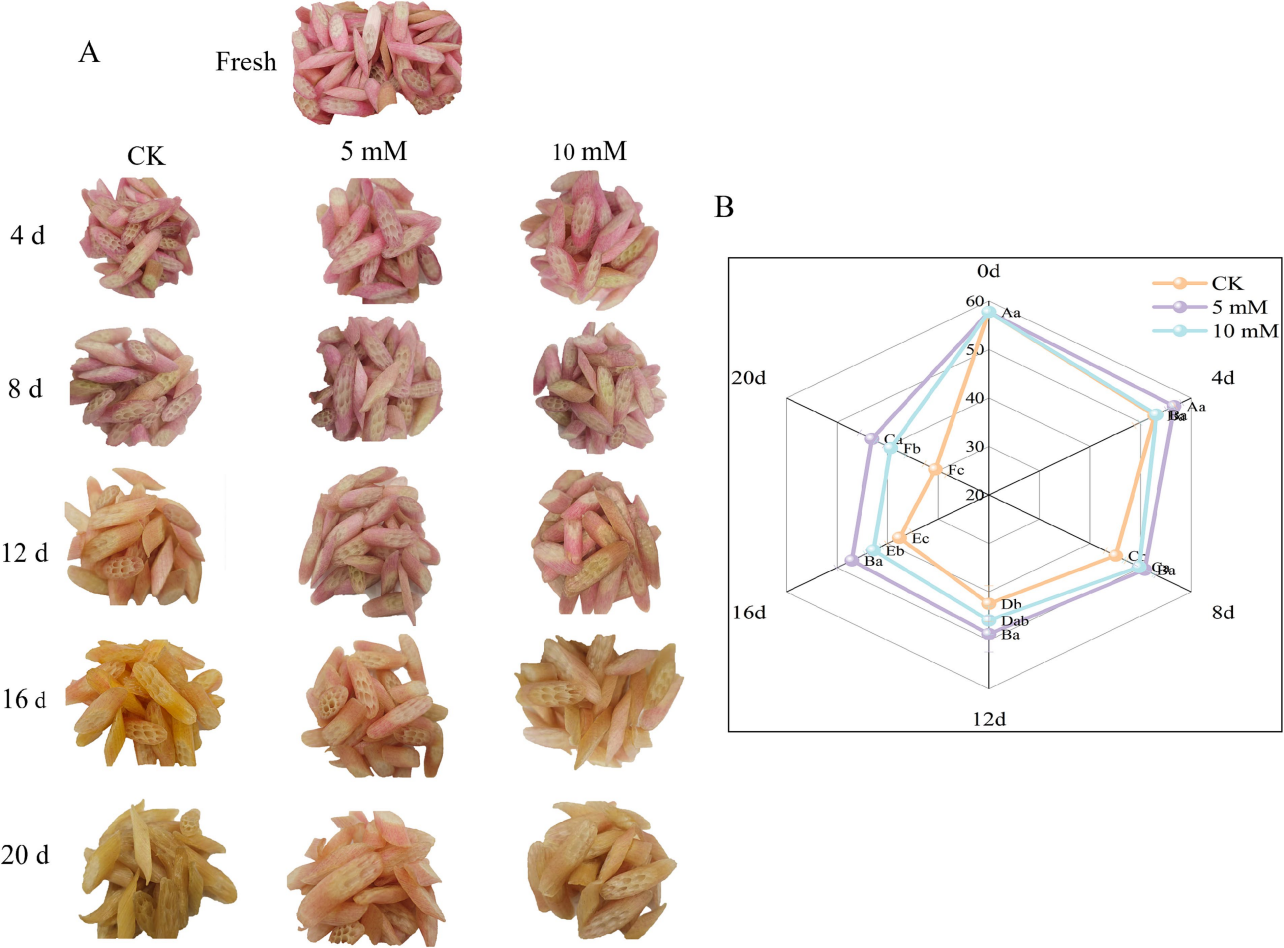
Effects of different concentrations of GABA on the overall appearance **(A)** and sensory quality **(B)** of FCEFS during storage at 4°C and 85–90 RH. Values with different lowercase letters at the same time point are significantly different (*p* < 0.05) between treatments. Values with different uppercase letters represent significant differences (*p* < 0.05) within a treatment at different sampling time points.

After the FCEFS samples were treated with different concentrations of GABA, the stems were photographed under storage conditions at 4°C. The results are shown in Figure 3A. After 20 d of storage, the colour of the FCEFS samples treated with varying concentrations of GABA changed from light red to cinerous. The control group showed rotting and water loss had no commercial value from 16 d. Notably, the 5 mM GABA-treated stems experienced the least water loss and presented the smallest colour change. These findings indicate that GABA treatment can reduce water loss in FCEFS and prolong its shelf-life.

To further evaluate the effects of different GABA concentrations on the quality of FCEFS during storage, the sensory quality was assessed (Figure 3B). The sensory quality of the stems decreased with increasing storage time. After 20 d of storage, the control group had the lowest sensory score of 30.56 ± 2.24 points, while the 5 mM GABA-treated group had the highest sensory score of 43.13 ± 2.48 points.

**Figure 4:**
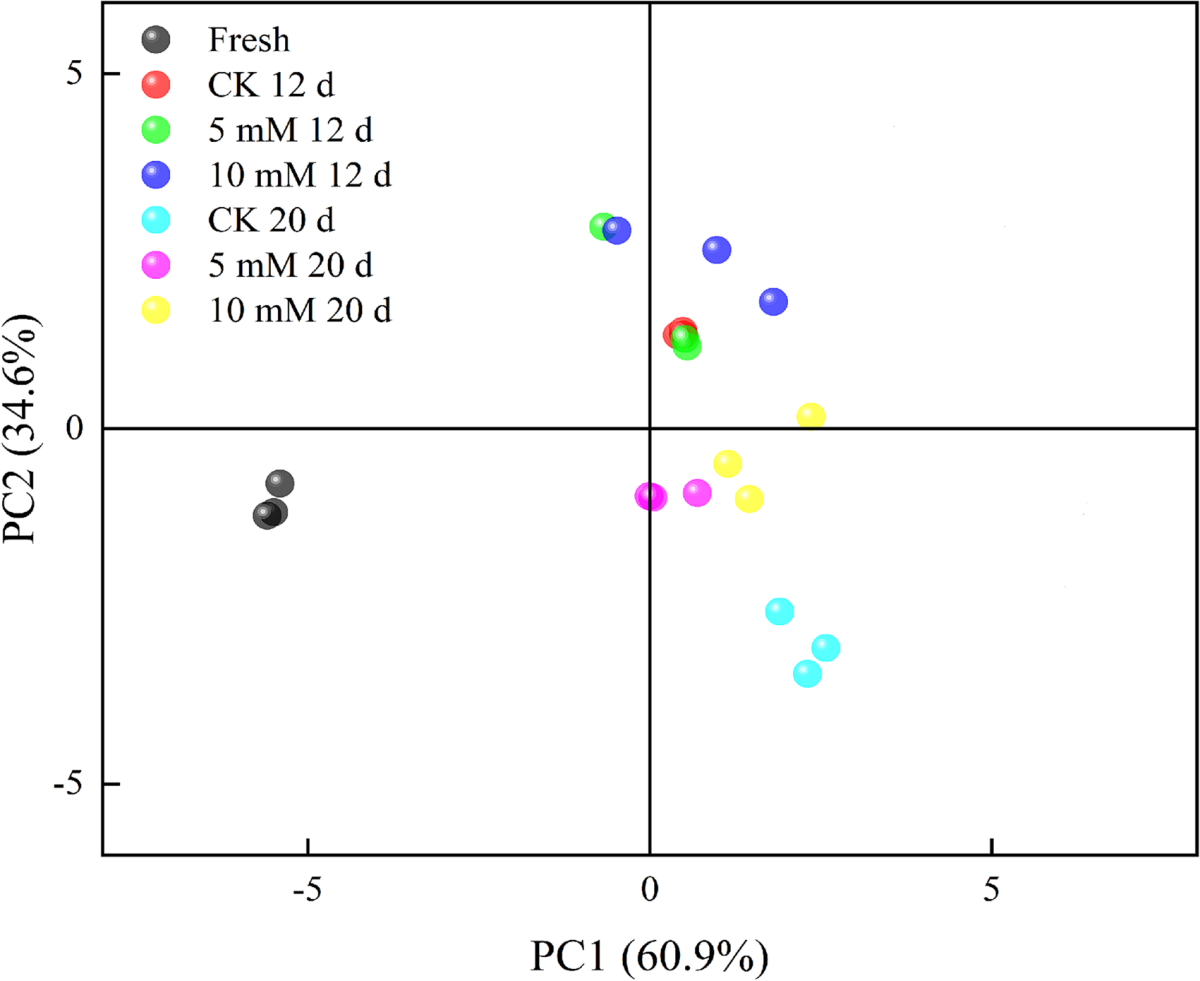
Principal component analysis (PCA) of ten volatile compounds (aromatic compounds, broad-range compounds, ammonium hydroxide, hydrogen, aromatic aliphatics, broad-range methane compounds, organosulfur compounds, broad-range alcohols, sulfur chlorinates, and methane aliphatics) measured in the FCEFS after 0, 12 and 20 d of storage at 4 ± 1°C with an electronic nose.

An electronic nose sensor array was created using an appropriate gas sensor to analyse how the FCEFS changed before and after storage (Figure 4). The smell analysis module primarily uses PCA to provide a more intuitive representation of the study results. Most of the gaseous components of the FCEFS were made up of broad-range compounds, broad-range methane compounds and organosulfur compounds. Figure 4 shows the PCA plot of the electronic nose data. PC1 accounted for 60.9% of the variation, PC2 accounted for 34.6%, and the total contribution rate was 95.5%, indicating that the experimental method was feasible and that the two principal components contained most of the information about the volatile odourant compounds in the samples. The odour contribution value of the 5 mM FCEFS treatment was close to that of the fresh sample stored for 20 d, indicating that the 5 mM GABA treatment is beneficial for storage. The middle-term storage of FCEFS revealed that the data points collected from the GABA treatment and the control group clustered together for the major portion of the storage. However, in the latter stage of storage after 20 d, a clear separation from the treatment data cluster was evident in the control. The results of the study showed changes in the FCEFS odour in the treatment groups, with a high degree of discreteness.

### 3.3 TSS, chlorophyll, and AsA contents

**Figure 5:**
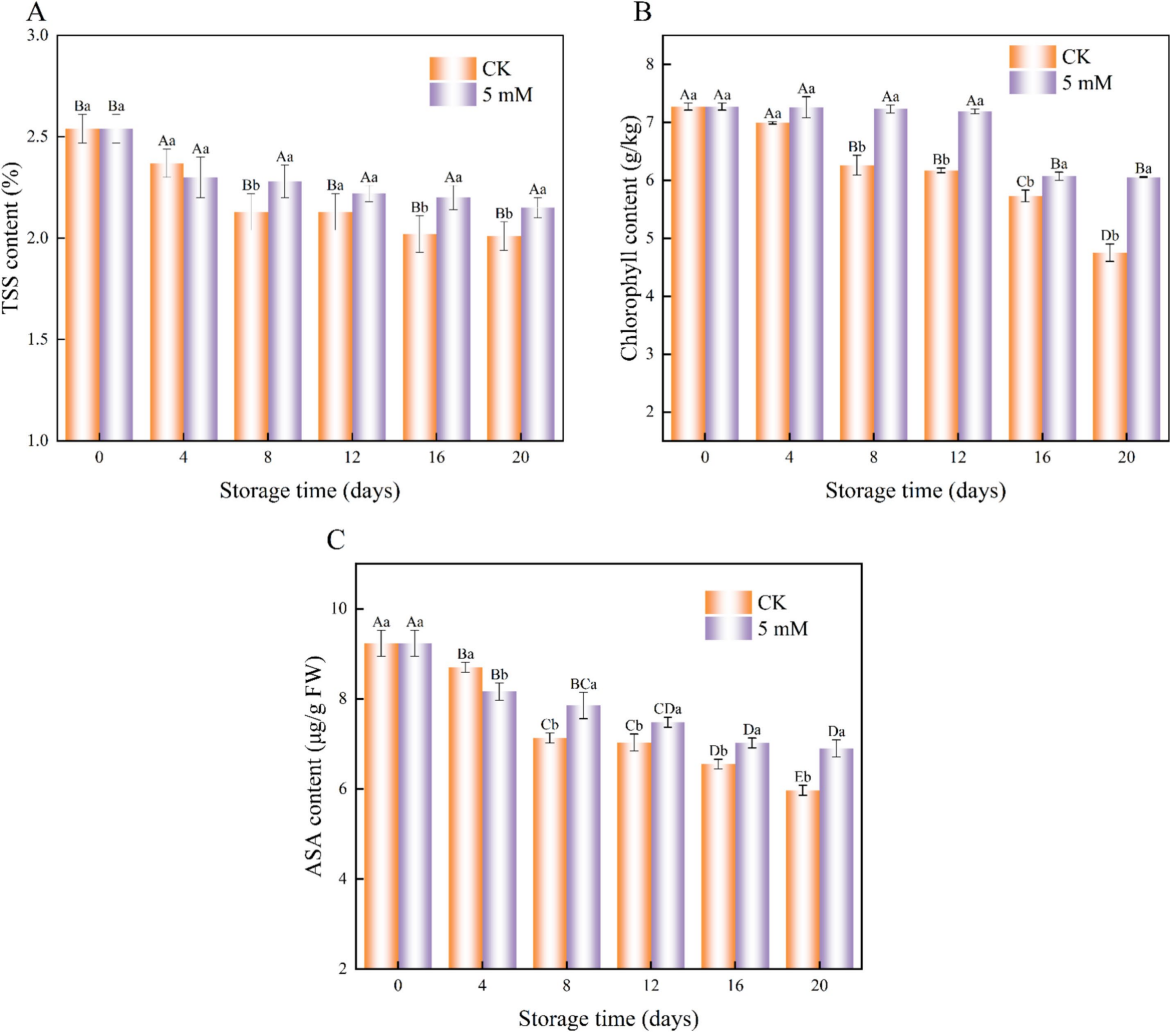
Interaction effects of the postharvest GABA content and storage time on the TSS content **(A)**, chlorophyll content **(B)** and ASA content **(C)** of the FCEFS stored at 4°C and 85–90 RH. Values with different lowercase letters at the same time point are significantly different (*p* < 0.05) between treatments. Values with different uppercase letters represent significant differences (*p* < 0.05) within a treatment at different sampling time points.

The effect of GABA treatment on FCEFS is shown in Figure 5. The TSS content of the FCEFS samples decreased gradually with increasing storage time (Figure 5A). Throughout the storage period, the TSS content of the GABA-treated group was greater than that of the control group. Significant differences in the TSS content were detected between the control group and the treated group at 20 d (*p* < 0.05).

During storage, the chlorophyll content of the FCEFS group gradually decreased, with the control group showing consistently lower chlorophyll levels than the GABA-treated group (Figure 5B). At 20 d, the chlorophyll content of the GABA-treated stems was 6.05 g/kg, which was 1.27 times greater than that of the control stems. These findings indicate that 5 mM GABA treatment can inhibit chlorophyll degradation in FCEFS, thereby delaying senescence and yellowing.

The AsA content of the treated FCEFS samples remained relatively high throughout the storage period. The AsA content decreased from 9.23 to 5.97 μg/g in the GABA-treated stems and from 9.23 to 6.9 μg/g in the control stems during refrigeration (Figure 5C). The decrease in AsA content was delayed by GABA treatment compared with that in the control.

### 3.4 Total phenolic content and DPPH radical scavenging capacity

**Figure 6:**
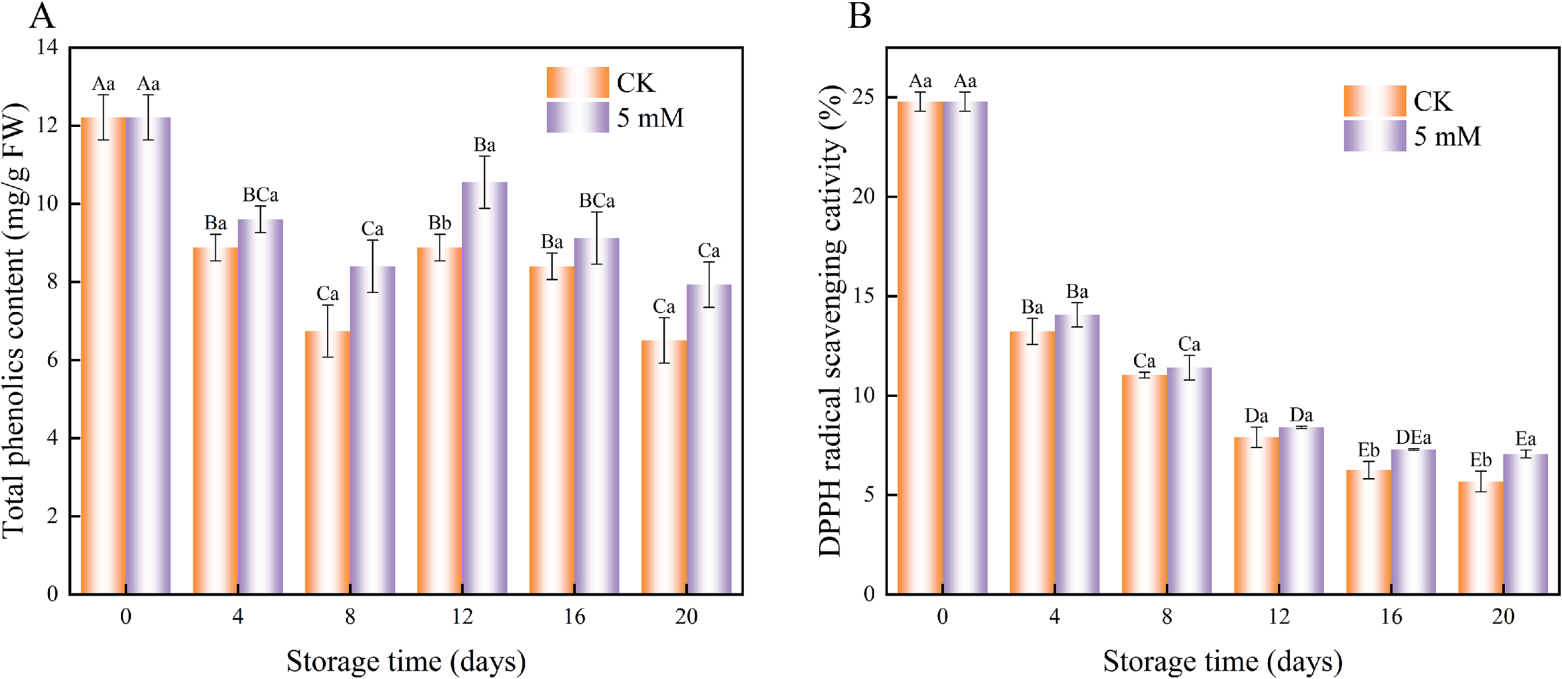
Interaction effect of the postharvest GABA content and storage time on the total phenolic content **(A)** and DPPH radical scavenging capacity **(B)** of the FCEFS samples stored at 4°C and 85–90% RH. Values with different lowercase letters at the same time point are significantly different (*p* < 0.05) between treatments. Values with different uppercase letters represent significant differences (*p* < 0.05) within a treatment at different sampling time points.

In this study, the total phenolic content of FCEFS was affected by changes in the control and GABA treatments with increasing storage time. The total phenolic content of the GABA-treated and control samples tended to decrease during storage (Figure 6A). The total phenolic content was greater in the GABA-treated samples than in the control samples during storage. This study demonstrated that GABA treatment effectively retarded the decline in total phenolic content in FCEFS.

The results revealed that the storage period affected the DPPH radical scavenging capacity. After 20 d, the DPPH scavenging activity was 1.24 times greater in the GABA-treated samples than in the control samples (Figure 6B). Although the DPPH radical scavenging capacity of FCEFS tended to decrease during storage, there was no significant difference between the control and treated samples (*p* > 0.05).

### 3.5 MDA content, H_2_O_2_ content, lignin content and lignin staining

**Figure 7:**
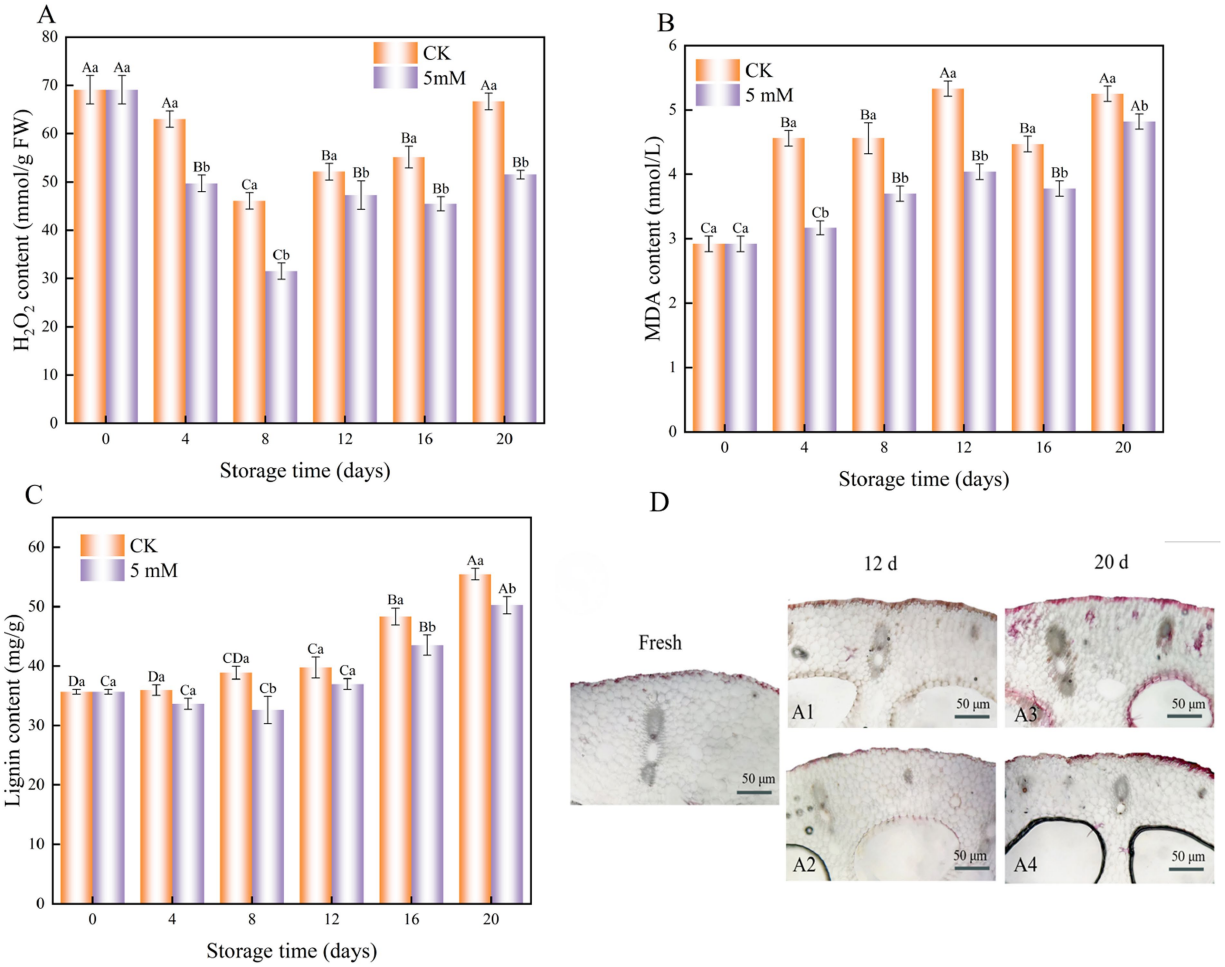
Interaction effects of the postharvest GABA content and storage time on the H_2_O_2_ content **(A)** MDA content **(B)** and lignin content **(C)** of FCEFS stored at 4°C and 85–90 RH. Light microscopy observations of the effect of GABA on lignin staining in FCEFS during cold storage **(D)**. (A1) CK, 12 d; (A2) 5 mM GABA, 12 d; (A3) CK, 20 d; (A4) 5 mM GABA, 20 d. Values with different lowercase letters at the same time point indicate significant differences (*p* < 0.05) between treatments. Values with different uppercase letters represent significant differences (*p* < 0.05) within a treatment at different sampling time points.

During the storage period, the H_2_O_2_ content in both the treatment and control groups initially decreased, followed by a gradual increase (Figure 7A). The lowest H₂O₂ content was measured on day 8, with H₂O₂ contents of 46.05 mmol/g and 31.51 mmol/g in the control and treatment groups, respectively. Notably, throughout the entire storage period, the H₂O₂ content in the treatment remained consistently and significantly lower than that in the control, with a highly significant difference (*p* < 0.05).

The MDA levels continuously increased from 0 to 20 d (Figure 7B). The greatest increase in MDA content was observed in the untreated FCEFS samples compared with the GABA-treated samples. After 20 d, the GABA-treated stems presented 1.09 times lower MDA levels than did the control stems. These findings indicate that GABA treatment significantly inhibited the accumulation of MDA during the postharvest storage of FCEFS.

Changes in lignin content during the storage of the FCEFS were observed. The results indicated an overall increase in lignin content during storage. The lignin content of the GABA-treated FCEFS was always lower than that of the control samples (Figure 7C). At the end of the 20 d of storage, the lignin content in the GABA-treated group was 1.10 times lower than that in the control group.

The results of lignin staining of FCEFS are shown in Figure 7D. Lignification had already begun after harvesting, but the degree of lignification was low, and it increased with increasing storage time after harvesting. The degree of lignin staining gradually increased from the outside to the inside, suggesting that lignin accumulated in the FCEFS from the outside to the inside. The results of lignin staining of the vascular bundles of FCEFS were consistent with of the results of lignin content measurements.

### 3.6 GSH, GR and endogenous GABA contents

**Figure 8:**
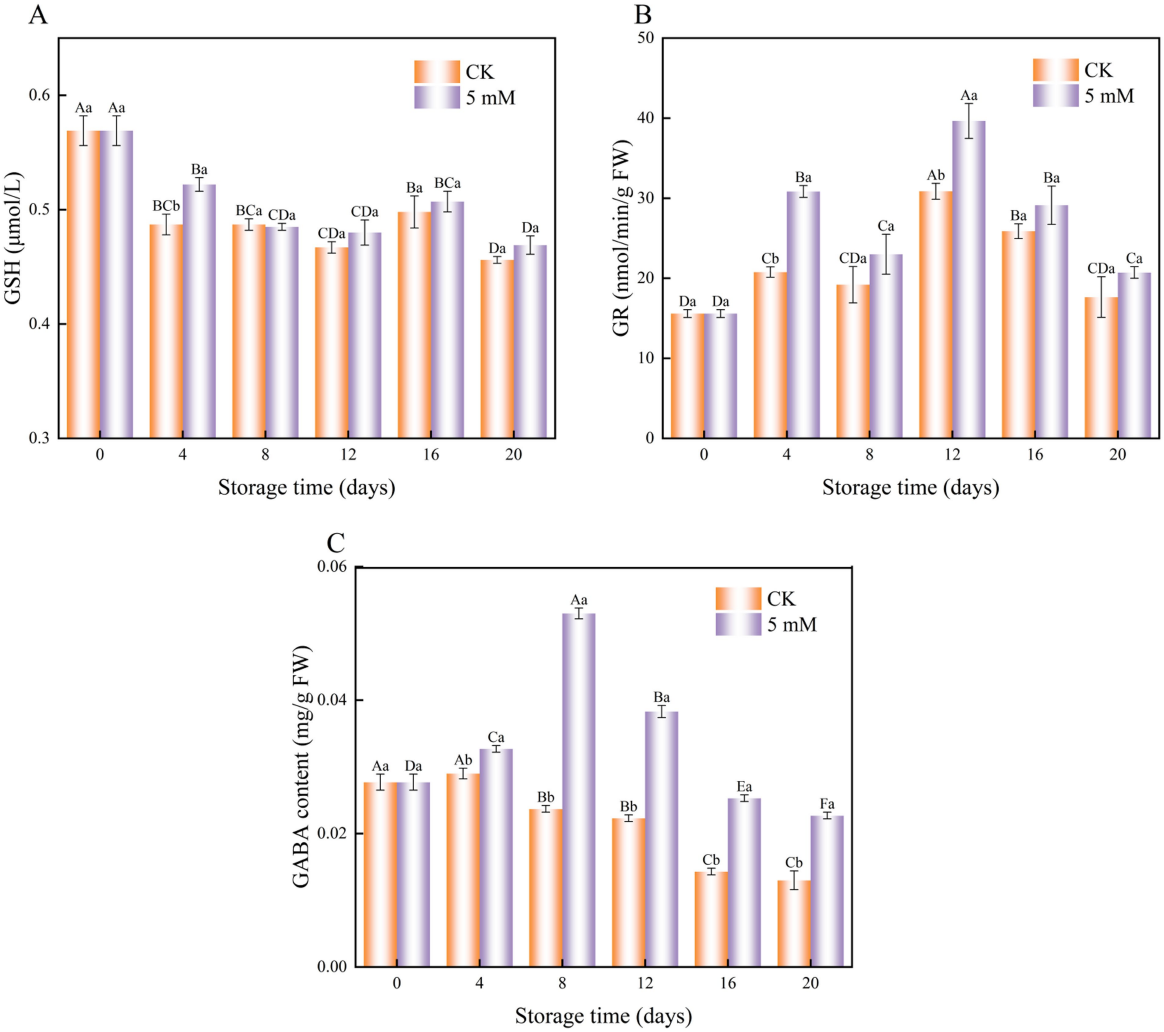
Interaction effect of postharvest GABA content and storage time on the GSH **(A)** GR **(B)** and endogenous GABA **(C)** contents of FCEFS stored at 4°C and 85–90 RH. Values with different lowercase letters at the same time point are significantly different (*p* < 0.05) between treatments. Values with different uppercase letters represent significant differences (*p* < 0.05) within a treatment at different sampling time points.

The results revealed a decrease in the GSH content of FCEFS (Figure 8A). The GSH content of the treatment group was 1.07 times greater than that of the control group on day 4 of cold storage. The GSH contents of the control group were 0.456 μmol/L and 0.469 μmol/L greater than that in the GABA treatment group after 20 d of cold storage.

GABA treatment exerted an effect on the GR activity of postharvest FCEFS during cold storage (Figure 8B). The GR activity in the treatment group was consistently greater than that in the control group. The greatest difference in GR content between the control and treatment groups was observed on day 12 of cold storage (*p* < 0.05), with the GR content of the treatment group being 1.28 times greater than that of the control.

The endogenous GABA content in GABA-treated FCEFS increased from 0–8 d and then decreased from 8–20 d (Figure 8C). GABA treatment significantly elevated the endogenous GABA content in FCEFS, with the highest level observed at 8 d. At this point, the GABA-treated stems presented an endogenous GABA content that was 2.24 times greater than that in the control.

### 3.7 POD activity, SOD activity, APX activity and PAL activity

**Figure 9:**
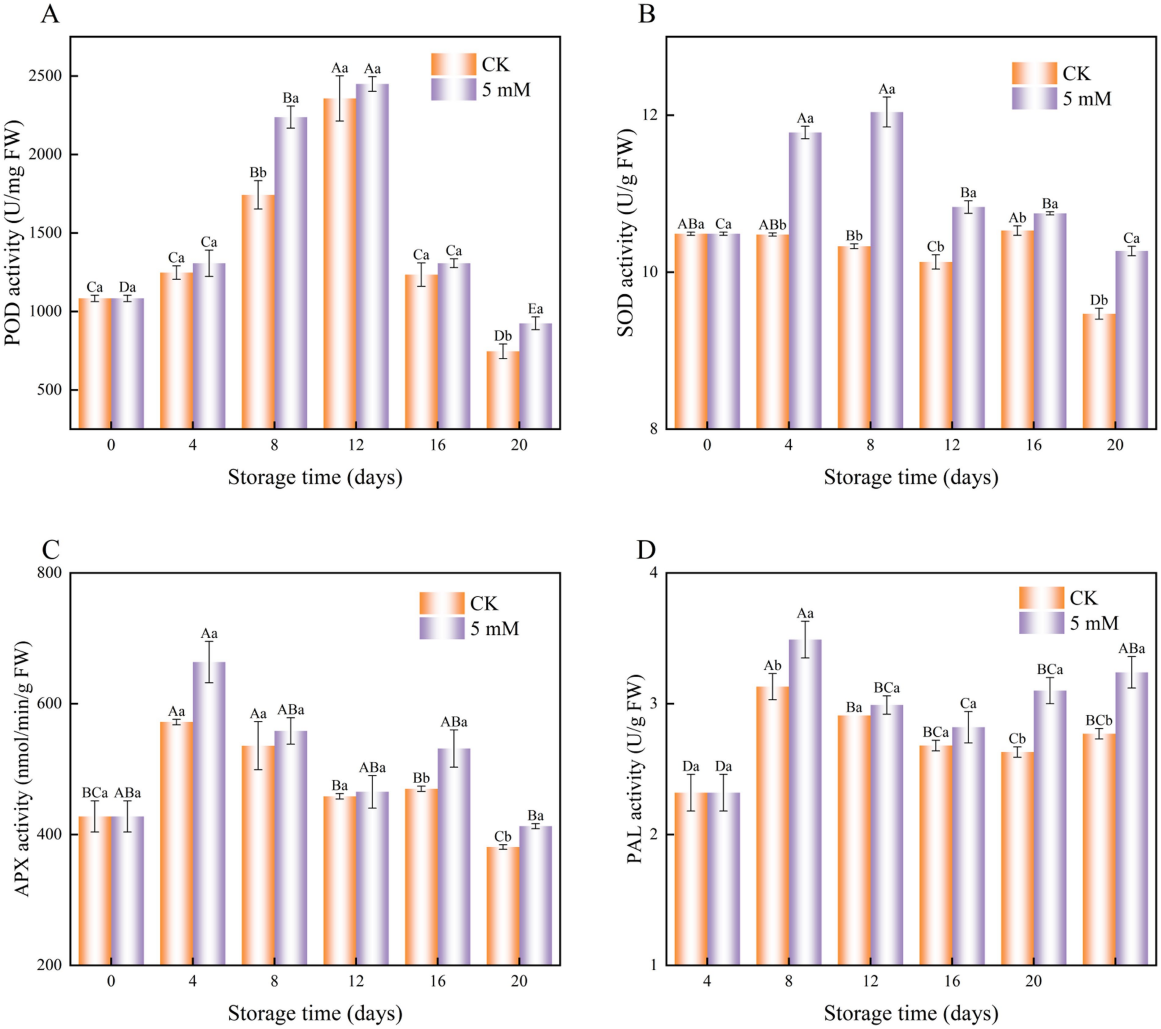
Interaction effect of postharvest GABA content and storage time on POD **(A)** SOD **(B)** APX **(C)** and PAL **(D)** activities in FCEFS stored at 4°C and 85–90 RH. Values with different lowercase letters at the same time point are significantly different (*p* < 0.05) between treatments. Values with different uppercase letters represent significant differences (*p* < 0.05) within a treatment at different sampling time points.

The POD activity of both the control and GABA-treated FCEFS samples tended to increase during the first 12 d, after which it gradually decreased until the end of the storage period (Figure 9A). However, the POD activity of the GABA-treated remained relatively high throughout the storage period.

SOD activity increased from 0–8 d posttreatment and then decreased in GABA-treated FCEFS (Figure 9B). GABA treatment significantly increased SOD activity from 0 to 12 d, with the highest level observed at 8 d posttreatment, which was 1.17 times greater than that in the control (*p* < 0.05).

The activity of APX in postharvest FCEFS varied significantly under GABA treatment throughout the storage period. The APX activity in the treatment group was consistently greater than that in control groups (Figure 9C). The APX activity in the treatment and the control group peaked on the fourth day of storage (571.99 and 663.70 nmol/min/g, respectively) and then decreased slowly.

GABA treatment also increased PAL activity in the FCEFS throughout the assay period. The highest PAL activity was detected in the GABA-treated stems at 4 d posttreatment, and the value was 1.11 times greater than that in the control stems (Figure 9D). During the storage period, the PAL activity in the control group was lower than that in the GABA treatment group.

### 3.8 Scanning electron microscopy analysis

**Figure 10:**
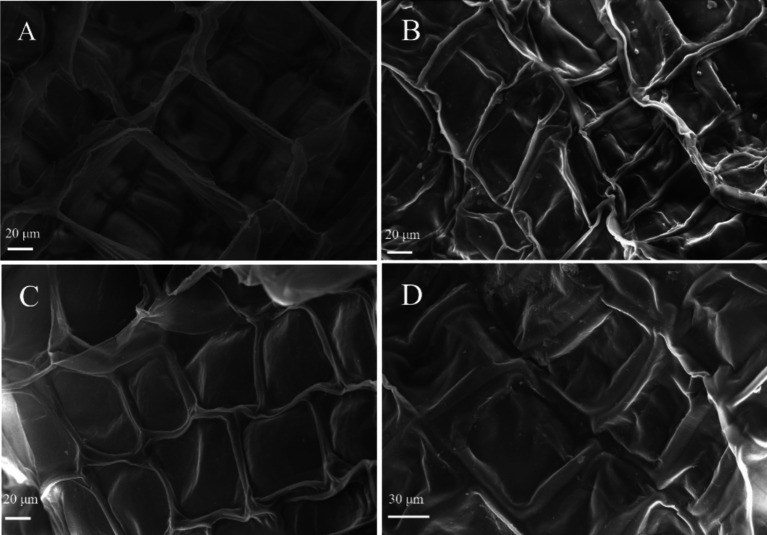
Effect of GABA treatment on the ultrastructure of FCEFS. **(A)** Fresh; **(B)** CK, 20 d; **(C)** 5 mM GABA; **(D)** 10 mM GABA. Magnification of the **A–D** images, 1,000x.

Scanning electron microscopy was used to analyse the ultrastructural changes in control and GABA-treated FCEFS tissues, and the results are shown in Figure 10. FCEFS cells were structurally intact, with smooth and tightly arranged cell walls. After 20 d of storage, changes were observed in the control tissue: the cell wall had collapsed and atrophied, with the complete structure no longer visible, and showing irregular, raised, and twisted features. These changes resulted in macroscopic signs of atrophy and softening, indicating obvious deterioration and autolysis. In contrast, the GABA-treated FCEFS samples appeared atrophied but relatively regular and flat, without the twisted appearance observed in the control after 20 d of storage. Cell wall atrophy and distortion were most pronounced in the control group. These results indicate that, compared with the control treatment, the 5 mM GABA treatment could delay the morphological changes in the FCEFS, reduce the disintegration rate and prolong the storage time.

### 3.9 Correlation analysis

**Figure 11:**
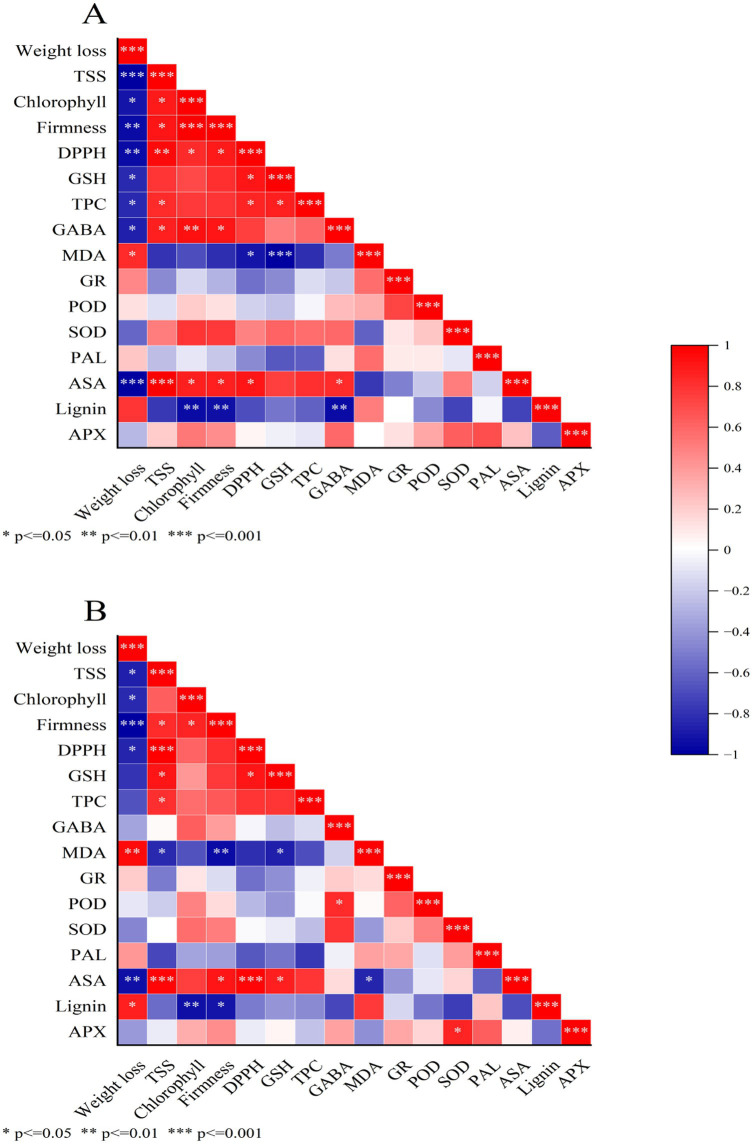
Heatmap of the results of the correlation analysis between the various parameters measured in FCEFS stored at 4°C for 20 d. Control FCEFS **(A)** FCEFS treated with GABA **(B)** Single asterisks (*) represent significant correlations at *p* < 0.05, double asterisks (**) represent significant correlations at *p* < 0.01, and three asterisks (***) represent significant correlations at *p* < 0.001. Red shading represents various degrees of positive correlations, and blue shading indicates various degrees of negative correlations, as indicated in the scale bar on the right of the heatmap.

The correlation between the parameters measured in this study was determined based on Pearson’s correlation coefficients (Figure 11). The correlation analysis indicated that weight loss in the control FCEFS samples was significantly negatively correlated with the TSS, chlorophyll, firmness, DPPH, GSH, TPC, GABA and ASA levels; among them, the correlation was most significant for the TSS and ASA contents. Weight loss was also positively correlated with the MDA content, GR content and lignin content (Figure 11A). The correlations in the GABA treatment and control groups were relatively similar. Notably, weight loss in the GABA-treated samples was most significantly negatively correlated with firmness (Figure 11B).

## 4 Discussion

### 4.1 Firmness, weight loss and colour

Fresh-cut fruits and vegetables are susceptible to weight loss during storage, primarily due to accelerated transpiration following fresh-cutting and processing. This leads to significant moisture loss, which affects the appearance, texture, flavour, and nutritional value of the produce and accelerates rotting and deterioration (24). The control sample presented the highest rate of mass loss during storage. In contrast, treatment with 5 mM GABA reduced the mass loss of FCEFS during cold storage. GABA, a small-molecule osmoregulator, can lower the osmotic water pressure in the cytoplasm and increase the water retention capacity of cells (25).

Firmness is a crucial indicator of the maturity and storage quality of fruits and vegetables. After harvest, the degradation of cell wall materials intensifies, reducing the viscosity between cells, thinning the cell walls, and relaxing their structure. This undermines the integrity of the cell wall, leading to softening of fruits and vegetables, and their firmness gradually decreases with maturity and ageing (26). These results suggest that GABA treatment effectively mitigated the firmness reduction. The application of GABA helps maintain cell membrane integrity, delays dehydration, and reduces firmness loss by slowing senescence and decreasing respiration and transpiration rates (27).

Oxidative browning and darkening of FCEFS were observed during cold storage. Studies on pear fruit (28), banana fruit (25), and other produce have demonstrated that GABA treatment effectively protects membrane integrity, as shown by lower electrolyte leakage and MDA content, thereby slowing browning during cold storage (29).

### 4.2 Appearance, sensory evaluation and electronic nose analyses

In general, consumers are most concerned about the colour, texture and flavour of vegetables when making purchases. The present study revealed that GABA treatment was effective in maintaining the stem quality and flavour of FCEFS. According to (4), the addition of GABA to pistachio nuts improves organoleptic attributes, including by reducing respiratory production, promoting retention of fruit moisture, and increasing antioxidant enzyme activity.

However, the GABA-treated FCEFS samples showed better parameters from the 4th day of storage than did the control samples, which slowly started to soften during the storage phase. These comparative observations were confirmed via principal component analysis of the electronic nose data.

### 4.3 TSS content, chlorophyll content and AsA content

In this study, the FCEFS samples treated with GABA maintained higher levels of nutrients such as TSS, chlorophyll and AsA than did the control samples. This increase can be attributed to biochemical alterations in the metabolism of the treated FCEFS samples, leading to increased retention of bioactive compounds. This, in turn, enhances the mechanism responsible for scavenging stress-generated free radicals, thereby maintaining the quality of the vegetable and increasing its market value (30). These results are consistent with those reported for sweet cherry (31) and loquat fruit (7).

### 4.4 Total phenolic content and DPPH radical scavenging capacity

FCEFS contains polyphenols, flavonoids, and other naturally occurring active substances, making it a good source of natural antioxidants. The antioxidant capacity of a tissue is indicated by metrics such as DPPH free radical scavenging activity.

Phenolic compounds are crucial secondary metabolites in plants that are essential for defending against postharvest pathogens in fruit and possess strong antioxidant activities that increase the nutritional value of the fruit. In this study, the total phenolic compound content and DPPH radical scavenging activity of the FCEFS samples were greater in the 5 mM GABA treatment group than in the control, which aligns with the findings of previous work (32).

### 4.5 MDA content, H_2_O_2_ content, lignin content and lignin staining

MDA is the end product of lipid peroxidation, which causes oxidative cellular damage and membrane dysfunction and plays a key role in promoting postharvest fruit senescence (33). Lipid peroxidation has been reported to increase in many plants under various environmental stresses (34). In the present study, the MDA content of FCEFS continued to increase after cold storage, suggesting that low-temperature stress leads to oxidative stress, which in turn results in the accumulation of large amounts of ROS, accelerating membrane lipid peroxidation and pericarp browning. Additionally, it was observed that the endogenous GABA content in subjected to exogenous GABA treatment differed from that in control. The results indicated that GABA treatment significantly inhibited the accumulation of MDA during the postharvest storage of FCEFS, alleviated the oxidative damage induced by lipid peroxidation, and improved the disease resistance of FCEFS, thereby delaying the postharvest senescence of FCEFS.

The excessive accumulation of reactive oxygen species (ROS) significantly contributes to the postharvest senescence of vegetables. The loss of water in fruits and vegetables postharvest can lead to cellular damage and plasmid degradation, resulting in tissue injury. Injured tissues can result in an elevated respiratory rate, which may subsequently exacerbate tissue damage (35). This study demonstrated that exogenous GABA treatment increased antioxidant enzyme activities and the antioxidant response to abiotic stress, reduced the accumulation of H_2_O_2_ and MDA, and protected the stability of the cell membrane structure.

Lignin, a product of plant lignification, is the primary component of the secondary structure of the cell wall. It hardens the cell wall and enhances cellular resistance to pressure in ligneous tissue (36). Under the action of the phenylpropanoid metabolic pathway, the healing tissue produces large amounts of monophenols, which are acted upon by related enzymes to form polyphenol cork lipids. Monophenols also participate in the lignin-specific metabolic pathway, synthesizing large quantities of lignin that accumulate around the healing tissue (37). Therefore, the 5 mM GABA treatment effectively inhibited lignin synthesis during the late storage period.

### 4.6 GSH, GR and endogenous GABA contents

GABA maintains the antioxidant capacity of FCEFS tissues by delaying the reduction in GSH and GR contents, thus preserving tissue nutrition and delaying senescence. GR and GSH are vital antioxidant agents in postharvest fruits and play crucial roles in the nonenzymatic ROS-scavenging system. GR is particularly important in the oxidative stress response and catalyses the reduction of GSSG by NADPH to generate GSH (8). H_2_O_2_ scavenging by APX is accompanied by the consumption of AsA, which results in the production of dehydroascorbate. GR then uses glutathione to regenerate ascorbic acid from dehydroascorbate (38).

GABA is involved in various stress responses as an intermediate in glutamate production within the plant GABA metabolic pathway. The prestorage application of GABA generally increases its endogenous concentration (39). Studies have also shown that exogenous GABA promotes the accumulation of endogenous GABA in fruits such as bananas (40) and peaches (25).

### 4.7 POD activity, SOD activity, APX activity and PAL activity

Although moderate amounts of ROS play important roles as signalling molecules in pathogen resistance and cellular signalling, excessive amounts of ROS cause oxidative damage and are deleterious to cells (41). Plants have evolved various enzymatic and nonenzymatic antioxidant mechanisms to prevent the accumulation of ROS. Enzymes such as POD, APX and SOD are designed to combat oxidative damage and scavenge ROS from plants. During the postharvest storage of fruits and vegetables, respiratory metabolism intensifies, energy metabolism diminishes, and ROS metabolism is exacerbated. The activity of antioxidant enzymes increases, and nonenzymatic antioxidants such as carotenoids, ascorbic acid, flavonols, and flavonoids work synergistically to form antioxidant defence systems (42). Antioxidant enzymes inhibit oxidative damage in fruits and vegetables by eliminating excessive ROS and harmful metabolites such as MDA, maintaining balanced levels of ROS. As vegetables continue to senesce, ROS levels increase, oxidative damage intensifies, and the activities of POD, APX, and SOD decrease. Consequently, the ability of the fruit to remove ROS decreases, leading to metabolic disorders and further senescence, ultimately resulting in death. GABA treatment has been shown to improve the postharvest performance of citrus fruits by increasing the activity of ROS scavenging enzymes such as SOD and CAT due to increased citrate accumulation and sufficient cellular ATP (43).

### 4.8 SEM analysis

Scanning electron microscopy can be utilized to examine the surface morphology of the tissue, the smoothness of cross-sections, cell morphology, and the impact of different treatments on the quality of fresh-cut fruits and vegetables during storage. FCEFS gradually senesces with prolonged storage time, and the most common deterioration at the end of the storage period is softening and rotting of the tissues and micromorphological deterioration of the structure and cellular morphology, while the most obvious feature is autolysis of the cells (44). The SEM revealed that 5 mM GABA treatment relatively maintained the integrity of the cell wall and reduced the occurrence of autolysis, which promoted the softening and senescence of FCEFS.

## 5 Conclusion

In addition, effective treatments are necessary to prevent spoilage and maintain the quality of the FCEFS. This research demonstrates that GABA has the potential to preserve the overall quality of FCEFS. The application of GABA inhibited lipid peroxidation and ROS production while maintaining the structural integrity of the cell membrane. The inhibition of lignin synthesis results in the slowing of cell wall metabolism and the lignification process. GABA treatment maintained high levels of nonenzymatic antioxidant components (AsA and total phenolics) and antioxidant enzyme activities (SOD, POD and APX), thereby alleviating the softening and senescence of postharvest FCEFS. GABA treatment sustained elevated levels of reduced GR and GSH, while inhibiting the accumulation of MDA and H_2_O_2_ in FCEFS. Additionally, GABA treatment helped maintain better organoleptic quality and mitigated the degradation of cellular structures. Furthermore, although studies have contributed to the understanding of the physiological mechanisms by which GABA treatment delays the softening of FCEFS, the underlying molecular mechanisms remain unknown. Consequently, additional studies are needed on the molecular mechanisms underlying the effects of GABA on the regulation of softening and ripening in *Euryale ferox* stems.

## Data Availability

The original contributions presented in the study are included in the article/supplementary material, further inquiries can be directed to the corresponding author.

## References

[1] Zhang W Jiang H Cao J Jiang W. Advances in biochemical mechanisms and control technologies to treat chilling injury in postharvest fruits and vegetables. Trends Food Sci Technol. (2021) 113:355–65. doi: 10.1016/j.tifs.2021.05.009

[2] Ali S Anjum MA Nawaz A Ejaz S Anwar R Khaliq G. Postharvest γ-aminobutyric acid application mitigates chilling injury of aonla (Emblica officinalis Gaertn.) fruit during low temperature storage. Postharvest Biol Technol. (2022) 185:111803. doi: 10.1016/j.postharvbio.2021.111803

[3] Xie H Shi G Wang R Chen Q Yu A Lu A. Euryale ferox stem-inspired anisotropic quaternized cellulose/xanthan-based antibacterial sponge with high absorbency and compressibility for noncompressible hemorrhage. Int J Biol Macromol. (2023) 237:124166. doi: 10.1016/j.ijbiomac.2023.124166 36965567

[4] Jalali H Nazoori F Mirdehghan SH Karimi HR. Pre-harvest application of GABA and CaO delays senescence and maintains of physicochemical characteristics of fresh ‘“Ahmad Aghaei”’ pistachio during cold storage. J Stored Prod Res. (2023) 104:102206. doi: 10.1016/j.jspr.2023.102206

[5] Zhu J Li C Sun L Cheng Y Hou J Fan Y. Application of γ-aminobutyric acid induces disease resistance in apples through regulation of polyamine metabolism, GABA shunt and reactive oxygen species metabolism. Sci Hortic. (2022) 291:110588. doi: 10.1016/j.scienta.2021.110588

[6] Shekari A Naghshiband Hassani R Soleimani AM. Exogenous application of GABA retards cap browning in Agaricus bisporus and its possible mechanism. Postharvest Biol Technol. (2021) 174:111434. doi: 10.1016/j.postharvbio.2020.111434

[7] Zhang H Pu J Liu H Wang M Du Y Tang X. Effects of L-cysteine and γ-aminobutyric acid treatment on postharvest quality and antioxidant activity of loquat fruit during storage. Int J Mol Sci. (2023) 24:10541. doi: 10.3390/ijms241310541, PMID: 37445735 PMC10341882

[8] Liu Q Li X Jin S Dong W Zhang Y Chen W. γ-Aminobutyric acid treatment induced chilling tolerance in postharvest kiwifruit (*Actinidia chinensis* cv. Hongyang) via regulating ascorbic acid metabolism. Food Chem. (2023) 404:134661. doi: 10.1016/j.foodchem.2022.134661, PMID: 36283321

[9] Duangmal K Saicheua B Sueeprasan S. Colour evaluation of freeze-dried roselle extract as a natural food colorant in a model system of a drink. LWT Food Sci Technol. (2008) 41:1437–45. doi: 10.1016/j.lwt.2007.08.014

[10] Jia B Zheng Q Zuo J Gao L Wang Q Guan W. Application of postharvest putrescine treatment to maintain the quality and increase the activity of antioxidative enzyme of cucumber. Sci Hortic. (2018) 239:210–5. doi: 10.1016/j.scienta.2018.05.043

[11] Zhu D Ren X Wei L Cao X Ge Y Liu H. Collaborative analysis on difference of apple fruits flavour using electronic nose and electronic tongue. Sci Hortic. (2020) 260:108879. doi: 10.1016/j.scienta.2019.108879

[12] Chen H Zhang M Guo Z. Discrimination of fresh-cut broccoli freshness by volatiles using electronic nose and gas chromatography-mass spectrometry. Postharvest Biol Technol. (2019) 148:168–75. doi: 10.1016/j.postharvbio.2018.10.019

[13] Ali S Khan AS Malik AU Shahid M. Effect of controlled atmosphere storage on pericarp browning, bioactive compounds and antioxidant enzymes of litchi fruits. Food Chem. (2016) 206:18–29. doi: 10.1016/j.foodchem.2016.03.021, PMID: 27041293

[14] García-Pastor ME Serrano M Guillén F Giménez MJ Martínez-Romero D Valero D. Preharvest application of methyl jasmonate increases crop yield, fruit quality and bioactive compounds in pomegranate ‘Mollar de Elche’ at harvest and during postharvest storage. J Sci Food Agric. (2020) 100:145–53. doi: 10.1002/jsfa.10007, PMID: 31471914

[15] Luo Q Tang Z Zhang X Zhong Y Yao S Wang L. Chemical properties and antioxidant activity of a water-soluble polysaccharide from Dendrobium officinale. Int J Biol Macromol. (2016) 89:219–27. doi: 10.1016/j.ijbiomac.2016.04.067 27131730

[16] Li N Chen F Cui F Sun W Zhang J Qian L. Improved postharvest quality and respiratory activity of straw mushroom (Volvariella volvacea) with ultrasound treatment and controlled relative humidity. Sci Hortic. (2017) 225:56–64. doi: 10.1016/j.scienta.2017.06.057

[17] Chumyam A Shank L Faiyue B Uthaibutra J Saengnil K. Effects of chlorine dioxide fumigation on redox balancing potential of antioxidative ascorbate-glutathione cycle in ‘Daw’ longan fruit during storage. Sci Hortic. (2017) 222:76–83. doi: 10.1016/j.scienta.2017.05.022

[18] Hu X Xu Z Xu W Li J Zhao N Zhou Y. Application of γ-aminobutyric acid demonstrates a protective role of polyamine and GABA metabolism in muskmelon seedlings under ca(NO3)2 stress. Plant Physiol Biochem. (2015) 92:1–10. doi: 10.1016/j.plaphy.2015.04.006, PMID: 25885476

[19] Terefe NS Tepper P Ullman A Knoerzer K Juliano P. High pressure thermal processing of pears: effect on endogenous enzyme activity and related quality attributes. Innov Food Sci Emerg Technol. (2016) 33:56–66. doi: 10.1016/j.ifset.2015.12.001

[20] Pan Y Chen L Pang L Chen X Jia X Li X. Ultrasound treatment inhibits browning and improves antioxidant capacity of fresh-cut sweet potato during cold storage. RSC Adv. (2020) 10:9193–202. doi: 10.1039/C9RA06418D, PMID: 35497218 PMC9050142

[21] Shi J Gao L Zuo J Wang Q Wang Q Fan L. Exogenous sodium nitroprusside treatment of broccoli florets extends shelf life, enhances antioxidant enzyme activity, and inhibits chlorophyll-degradation. Postharvest Biol Technol. (2016) 116:98–104. doi: 10.1016/j.postharvbio.2016.01.007

[22] Toscano S Rizzo V Licciardello F Romano D Muratore G. Packaging solutions to extend the shelf life of green Asparagus (*Asparagus officinalis* L.) 'Vegalim'. Food Secur. (2021) 10:478. doi: 10.3390/foods10020478, PMID: 33671803 PMC7926684

[23] Chi H Lu W Liu G Qin Y. Physiochemical property changes and mineral element migration behavior of bamboo shoots during traditional fermentation process. J Food Process Preserv. (2020) 44:E14784. doi: 10.1111/jfpp.14784

[24] Lin Y Lin Y Lin Y Lin M Chen Y Wang H. A novel chitosan alleviates pulp breakdown of harvested longan fruit by suppressing disassembly of cell wall polysaccharides. Carbohydr Polym. (2019) 217:126–34. doi: 10.1016/j.carbpol.2019.04.053, PMID: 31079668

[25] Wang Y Luo Z Huang X Yang K Gao S Du R. Effect of exogenous γ-aminobutyric acid (GABA) treatment on chilling injury and antioxidant capacity in banana peel. Sci Hortic. (2014) 168:132–7. doi: 10.1016/j.scienta.2014.01.022

[26] Chen H Cao S Fang X Mu H Yang H Wang X. Changes in fruit firmness, cell wall composition and cell wall degrading enzymes in postharvest blueberries during storage. Sci Hortic. (2015) 188:44–8. doi: 10.1016/j.scienta.2015.03.018

[27] Xiong S Sun X Tian M Xu D Jiang A. 1-Methylcyclopropene treatment delays the softening of *Actinidia arguta* fruit by reducing cell wall degradation and modulating carbohydrate metabolism. Food Chem. (2023) 411:135485. doi: 10.1016/j.foodchem.2023.135485, PMID: 36682166

[28] Rabiei V Kakavand F Zaare-Nahandi F Razavi F Aghdam MS. Nitric oxide and γ-aminobutyric acid treatments delay senescence of cornelian cherry fruits during postharvest cold storage by enhancing antioxidant system activity. Sci Hortic. (2019) 243:268–73. doi: 10.1016/j.scienta.2018.08.034

[29] Li J Zhou X Wei B Cheng S Zhou Q Ji S. GABA application improves the mitochondrial antioxidant system and reduces peel browning in ‘Nanguo’ pears after removal from cold storage. Food Chem. (2019) 297:124903. doi: 10.1016/j.foodchem.2019.05.177, PMID: 31253345

[30] Sharma S Krishna H Barman K Kole B Singh SK Behera TK. Synergistic effect of polyamine treatment and chitosan coating on postharvest senescence and enzyme activity of bell pepper (*Capsicum annuum* L.) fruit. South Afr J Bot. (2022) 151:175–84. doi: 10.1016/j.sajb.2022.09.002

[31] Carrión-Antolí A Badiche-El Hilali F Lorente-Mento JM Díaz-Mula HM Serrano M Valero D. Antioxidant systems and quality in sweet cherries are improved by Preharvest GABA treatments leading to delay postharvest senescence. Int J Mol Sci. (2023) 25:260. doi: 10.3390/ijms25010260, PMID: 38203428 PMC10779314

[32] Ebrahimzadeh A Pirzad F Tahanian H Aghdam MS. Influence of gum Arabic enriched with GABA coating on oxidative damage of walnut kernels. Food Technol Biotechnol. (2019) 57:554–60. doi: 10.17113/ftb.57.04.19.6380, PMID: 32123517 PMC7029382

[33] Li D Limwachiranon J Li L Du R Luo Z. Involvement of energy metabolism to chilling tolerance induced by hydrogen sulfide in cold-stored banana fruit. Food Chem. (2016) 208:272–8. doi: 10.1016/j.foodchem.2016.03.113 27132850

[34] Pucciariello C Banti V Perata P. ROS signaling as common element in low oxygen and heat stresses. Plant Physiol Biochem. (2012) 59:3–10. doi: 10.1016/j.plaphy.2012.02.016, PMID: 22417734

[35] Ji N Wang J Zuo X Li Y Li M Wang K. PpWRKY45 is involved in methyl jasmonate primed disease resistance by enhancing the expression of jasmonate acid biosynthetic and pathogenesis-related genes of peach fruit. Postharvest Biol Technol. (2021) 172:111390. doi: 10.1016/j.postharvbio.2020.111390

[36] Hong P Zhang J Shi D Yang C Zeng M Li X. Postharvest application of methyl jasmonate alleviates lignin accumulation in stone cells of pear fruit during low-temperature storage. Postharvest Biol Technol. (2024) 209:112692. doi: 10.1016/j.postharvbio.2023.112692

[37] Vanholme R De Meester B Ralph J Boerjan W. Lignin biosynthesis and its integration into metabolism. Curr Opin Biotechnol. (2019) 56:230–9. doi: 10.1016/j.copbio.2019.02.018, PMID: 30913460

[38] Soleimani Aghdam M Naderi R Jannatizadeh A Sarcheshmeh MAA Babalar M. Enhancement of postharvest chilling tolerance of anthurium cut flowers by γ-aminobutyric acid (GABA) treatments. Sci Hortic. (2016) 198:52–60. doi: 10.1016/j.scienta.2015.11.019

[39] Shang H Cao S Yang Z Cai Y Zheng Y. Effect of exogenous γ-aminobutyric acid treatment on Proline accumulation and chilling injury in peach fruit after long-term cold storage. J Agric Food Chem. (2011) 59:1264–8. doi: 10.1021/jf104424z, PMID: 21287990

[40] Yang A Cao S Yang Z Cai Y Zheng Y. γ-Aminobutyric acid treatment reduces chilling injury and activates the defence response of peach fruit. Food Chem. (2011) 129:1619–22. doi: 10.1016/j.foodchem.2011.06.018

[41] Lin Y Lin Y Lin H Zhang S Chen Y Shi J. Inhibitory effects of propyl gallate on browning and its relationship to active oxygen metabolism in pericarp of harvested longan fruit. LWT Food Sci Technol. (2015) 60:1122–8. doi: 10.1016/j.lwt.2014.10.008

[42] Vall-llaura N Fernández-Cancelo P Nativitas-Lima I Echeverria G Teixidó N Larrigaudière C. ROS-scavenging-associated transcriptional and biochemical shifts during nectarine fruit development and ripening. Plant Physiol Biochem. (2022) 171:38–48. doi: 10.1016/j.plaphy.2021.12.022, PMID: 34971954

[43] Sheng L Shen D Luo Y Sun X Wang J Luo T. Exogenous γ-aminobutyric acid treatment affects citrate and amino acid accumulation to improve fruit quality and storage performance of postharvest citrus fruit. Food Chem. (2017) 216:138–45. doi: 10.1016/j.foodchem.2016.08.024, PMID: 27596402

[44] Xu D Zhou F Gu S Feng K Hu W Zhang J. 1-Methylcyclopropene maintains the postharvest quality of hardy kiwifruit (Actinidia aruguta). J Food Meas Charact. (2021) 15:3036–44. doi: 10.1007/s11694-021-00893-y

